# Maintenance energy requirements of odor detection, explosive detection and human detection working dogs

**DOI:** 10.7717/peerj.767

**Published:** 2015-02-24

**Authors:** Rebecca A. Mullis, Angela L. Witzel, Joshua Price

**Affiliations:** 1 Department of Small Animal Clinical Sciences, University of Tennessee College of Veterinary Medicine, Knoxville, TN, USA; 2 Office of Information Technology, University of Tennessee, Knoxville, TN, USA

**Keywords:** Canine, Nutrition, Metabolism, Energy, Dog, Veterinary, Exercise

## Abstract

Despite their important role in security, little is known about the energy requirements of working dogs such as odor, explosive and human detection dogs. Previous researchers have evaluated the energy requirements of individual canine breeds as well as dogs in exercise roles such as sprint racing. This study is the first to evaluate the energy requirements of working dogs trained in odor, explosive and human detection. This retrospective study evaluated twenty adult dogs who maintained consistent body weights over a six month period. During this time, the average energy consumption was $136\pm 38~\mathrm{kcal}\cdot {\mathrm{BW}}_{\mathrm{kg}}^{0.75}$ or two times the calculated resting energy requirement ($\mathrm{RER}=70~\mathrm{kcal}\cdot {\mathrm{BW}}_{\mathrm{kg}}^{0.75}$). No statistical differences were found between breeds, age or sex, but a statistically significant association (*p* = 0.0033, *R*-square = 0.0854) was seen between the number of searches a dog performs and their energy requirement. Based on this study’s population, it appears that working dogs have maintenance energy requirements similar to the 1974 National Research Council’s (NRC) maintenance energy requirement of $132~\mathrm{kcal}\cdot {\mathrm{BW}}_{\mathrm{kg}}^{0.75}$ (National Research Council (NRC), 1974) and the $139\pm 42~\mathrm{kcal}\cdot {\mathrm{BW}}_{\mathrm{kg}}^{0.75}$ reported for young laboratory beagles (Rainbird & Kienzle, 1990). Additional research is needed to determine if these data can be applied to all odor, explosive and human detection dogs and to determine if other types of working dogs (tracking, search and rescue etc.) have similar energy requirements.

## Introduction

Working dogs are an integral part of the security system in place in the United States. They are used in multiple locations such as secure military facilities and airports, and can serve a number of purposes such as human and explosive detection. While other security measures are in place in these facilities, dogs play an important role due to their tremendous odor sensing abilities and their willingness to be trained and perform. Shepherds including Belgian Malinois and Labrador retrievers are among the most common breeds used for these jobs. While dogs are frequently used in this role, no studies have been published documenting their energy needs.

In mammals, resting energy requirements (RER) can be calculated based on the equation RER (kcal/day) $=70~\mathrm{kcal}\cdot {\mathrm{BW}}_{\mathrm{kg}}^{0.75}$ (Kleiber, 1961) and reflects the minimal amount of energy required daily to maintain body weight in the absence of factors such as exercise or environmental temperatures which could increase energy needs. Maintenance energy requirements (MER) describe the amount of energy an animal needs to support energy equilibrium and accounts for thermoregulation, spontaneous activity and exercise. It also accounts for energy lost as heat during dietary thermogenesis or the metabolism and digestion of foods. In the scientific literature, MER established through research studies are often reported as $\mathrm{kcal}\cdot {\mathrm{BW}}_{\mathrm{kg}}^{0.75}$. In clinical practice, many nutritionists estimate maintenance energy requirements by applying a life stage factor to the calculated resting energy requirement (Thatcher, Hand & Remillard, 2010). A wide range of life stage factors are used depending on an animal’s needs such as weight loss or lactation. The (National Research Council (NRC), 1974) suggested that the average maintenance energy requirement of adult dogs in kennels undertaking moderate exercise is $132~\mathrm{kcal}\cdot {\mathrm{BW}}_{\mathrm{kg}}^{0.75}$ per day. More recent data looking at Great Danes, middle-aged Newfoundlands and racing greyhounds (Zentek & Meyer, 1992; Rainbird & Kienzle, 1990; Hill et al., 2000; Hinchcliff et al., 1997) have shown that this value is likely valid for young laboratory dogs but variation exists between other populations. A wide variation in maintenance energy requirements has been documented from 94 to $250~\mathrm{kcal}\cdot {\mathrm{BW}}_{\mathrm{kg}}^{0.75}$ per day (National Research Council (NRC), 2006).

While there has been no published research documenting energy requirements for working dogs, it has been estimated that low-activity service dogs have a small increase (<25%) in energy requirements from active dog maintenance energy requirements ($132~\mathrm{kcal}\cdot {\mathrm{BW}}_{\mathrm{kg}}^{0.75})$, and high-activity service dogs and search and rescue have a moderate increase (25%–100%) in energy requirements from active dog maintenance requirements (Wakshlag & Shmalberg, 2014). This retrospective study was designed to determine the maintenance energy requirements for working dogs with a focus on those dogs performing odor, human and explosive detection. Variables including age, breed, sex, number of hours worked, and the number of searches performed each month were analyzed to determine which factors altered their energy requirements.

## Materials and Methods

This retrospective study examined a population of 20 dogs (eleven intact females, nine intact males) that ranged in age from 3 years to 12 years of age with a mean age of 7.5 ± 2.4 years. Breeds represented included Border collie (*n* = 1), Belgian Malinois (*n* = 10), Dutch Shepherd (*n* = 1), German Shepherd Dog (*n* = 2) and Labrador retriever (*n* = 6). A list of the dogs with their corresponding age, sex, mean weights and weight ranges can be found in Table 1. These dogs were housed individually in indoor kennels. The indoor temperature of the housing facility was kept close to outdoor temperatures but never dropped below 7.2 °C or was higher than 32.2 °C. Average high and low outdoor temperatures at the kennel can be found in Table 2. All dogs in this study received routine medical care by a local veterinarian and were deemed healthy during the study period.

**Table 1:** Population data.

Dog	Breed	Age (years)	Sex	Mean weight (kg)	Weight range (kg)	Cups fed per day	Kcal offered per day
Dog 1	Belgian Malinois	10	Male	28.2	27.4–28.6	5	1734
Dog 2	Labrador Retriever	12	Male	29.7	29.1–30.0	6	2081
Dog 3	Belgian Malinois	10	Female	20.6	19.6–21.5	3.5	1214
Dog 4	German Shepherd Dog	8	Female	34.9	33.2–36.1	4	1387
Dog 5	Labrador Retriever	9	Male	30.4	29.8–31.5	5.5	1907
Dog 6	Belgian Malinois	8	Male	32.9	30.9–33.8	6	2081
Dog 7	Belgian Malinois	6	Female	17.9	17.6–18.2	2.5	867
Dog 8	Belgian Malinois	7	Male	27.9	26.6–29.0	4.5	1561
Dog 9	Border Collie	9	Male	23.4	23.0–23.6	4.5	1561
Dog 10	Belgian Malinois	7	Female	24.8	23.6–25.9	6	2081
Dog 11	Belgian Malinois	6	Female	26.5	25.0–28.2	6	2081
Dog 12	Belgian Malinois	6	Female	22.3	18.8–23.7	4	1387
Dog 13	Labrador Retriever	7	Female	27.4	25.5–29.2	3	1040
Dog 14	Belgian Malinois	6	Female	24.5	23.6–25.4	6	2081
Dog 15	Labrador Retriever	10	Male	34.6	33.7–35.7	3	1040
Dog 16	Labrador Retriever	10	Male	33.7	32.7–34.2	3.5	1214
Dog 17	Labrador Retriever	3	Male	30.8	29.7–31.4	3	1040
Dog 18	Dutch Shepherd	3	Female	25.1	23.6–26.4	6	2081
Dog 19	German Shepherd Dog	4	Female	28.7	27.3–30.1	7	2275
Dog 20	Belgian Malinois	9	Female	25.0	24.6–25.5	4	1387

**Table 2:** Average outdoor temperatures. (°C)

Month	Average high	Average low
May	25.6	12.4
June	27.9	18.9
July	29.3	19.6
August	29.8	19.6
September	27.7	16.3
October	22.1	10.0

These dogs were used for explosive, human and odor detection between May 2013 and October 2013. The dogs were each weighed once monthly by the kennel staff who utilized the same scale^1^1A and A Scale, LLC, Prospect Park, NJ, USA. at each weight check. The dogs were fed a commercial maintenance diet^2^2Eukanuba Canine Adult Maintenance 1 + (dry), P&G Pet Care, Cincinnati, OH, USA. except in the case of one dog that had food allergies and was fed a therapeutic hydrolyzed veterinary diet.^3^3Hill’s Prescription Diet^®^ *z*/*d*^®^ Canine Ultra (dry), Hill’s Pet Nutrition, Topeka, KS, USA. Macronutrient profiles of the two diets can be found in Table 3. All dogs in this study were fed either once or twice daily depending on their previous feeding schedules assigned by the kennel staff. The feeding amounts were determined by the kennel staff and the food was measured for each dog using a measuring cup. Daily feeding amounts for the individual dogs can be found in Table 1. All dogs included in this study consumed 100% of their offered food each day. The daily energy intake was calculated by multiplying the number of cups consumed per day by the caloric density of the diet as provided by the manufacturers. All dogs had free access to water. The dogs maintained body condition scores between a 4/9 and a 6/9 during the study period (Laflamme, 1997). The primary investigator evaluated each dog’s body condition to determine ideal body weights. Each dog’s ideal body weight was calculated using 20% body fat as ideal. The maintenance energy requirement was determined by observing the energy intake required to maintain the dogs close to their ideal weights. The number of hours worked for each dog was recorded by the dog handlers in 15 min increments and entered into a database at the kennel facility.

**Table 3:** Diet analysis.

	Maintenance diet		Hydrolyzed diet	
ME content	346.77 kcal/cup		325 kcal/cup	
	g/1,000 kcal	Guaranteed analysis	g/1,000 kcal	Guaranteed analysis
Protein	69.1	25%	48	14%
Fat	44.3	16%	33	10%
Carbohydrates/nitrogen free extracts	Not provided	38.5%	147	55.5%
Fiber	4.1	5%	7	5%
Ash	N/A	5.5%	N/A	5.5%
Moisture	N/A	10%	N/A	10%

Three statistical models were developed in SAS version 9.4 order to analyze the data. An initial mixed model was fit by performing a split plot repeated measures analysis of variance (ANOVA) on the rank transformed energy values. This model included two whole plot factors, one split plot factor, and three additional continuous covariates. The two whole plot factors were gender and breed, and the split plot factor was time. The three covariates were age, hours worked and number of searches. A rank transformation was used on the energy values for the initial model in order to help meet the underlying statistical assumptions required for split plot ANOVA designs. The rank transformation was not required in order to meet the assumptions of the 2 subsequent regression models. A *p*-value of <0.1 was considered significant for inclusion into the reduced model and a p-value of <0.05 was considered significant for inclusion in the final model.

## Results & Discussion

This study is the first to approximate the MER of working (odor, explosive and human detection) dogs and evaluate factors that could influence their energy requirements. The initial mixed model split-plot ANOVA Type III Tests for Fixed Effects demonstrated that number of hours a dog worked (*F* = 3.53, d.f. = 1,50.46, *p*-value = 0.0661) and number of searches performed (*F* = 5.32, d.f. = 1,50.22, *p*-value = 0.0253) could have a statistically significant impact on energy requirements. The dogs in this study did not perform strenuous work but were active and alert during their working hours. The mean hours worked each month was 132.8 ± 66.6 h and the mean number of searches (odor detection, vehicle, human) per month was 2,511 ± 3,232. A reduced multiple linear regression model was fit including both covariates. This resulted in a statistically significant result for the number of searches performed (*t* = − 3.02, d.f. = 1, *p*-value = 0.0033). However, the reduced model demonstrated that the number of hours worked was not statistically significant. A final simple linear regression model was fit and concluded that the number of searches a dog performs has a statistically significant effect on a dog’s energy level (*t* = − 3.01, d.f. = 1, *p*-value = 0.0033). All statistical assumptions regarding linearity, homoscedastic, normality of the residuals, independence of the error terms, and the lack of residual outliers were met. The final model demonstrates an *R*-square value of 0.0854. Therefore we can conclude that approximately 8.5% of a dog’s energy level can be explained by the number of searches a dog performs.

Working dogs in this study had an average energy intake of $135\pm 38~\mathrm{kcal}\cdot {\mathrm{BW}}_{\mathrm{kg}}^{0.75}$. While these dogs were usually close to their ideal body weights, monthly weight fluxuations were present. While this limitation was unavoidable due to the study’s retrospective nature, most dogs had less than a 6% deviation from their mean weight during any given month. All dogs except one had less than a 10% deviation from their mean weight during any given month. In dog 12, a 15.6% variation was noted during one month when a weight loss of 3.36 kgs was recorded. Human error is the suspected cause of this variation because the dog’s exercise level and food intake were consistent during this study, and the dog’s weight was 4.9 kgs higher when rechecked the following month. When evaluating MER using an ideal body weight, the average life stage factor used to approximate their daily energy requirement was 2.0 ± 0.5 with a range from 1.1 to 2.8.

The average MER of this group of dogs ($136\pm 38~\mathrm{kcal}\cdot {\mathrm{BW}}_{\mathrm{kg}}^{0.75}$) was similar to those of laboratory beagles with a mean age of 6.5 years who required $144\pm 28~\mathrm{kcal}\cdot {\mathrm{BW}}_{\mathrm{kg}}^{0.75}$ (Finke, 1994) and middle aged laboratory Labrador retrievers (range of 3–7 years) who required $136\pm 3.6~\mathrm{kcal}\cdot {\mathrm{BW}}_{\mathrm{kg}}^{0.75}$ (Kienzle & Rainbird, 1991) . In comparison, sled dogs who were more active and exposed to low environmental temperatures had a much higher requirement with MER ranging from $228~\mathrm{kcal}\cdot {\mathrm{BW}}_{\mathrm{kg}}^{0.75}$ to $\text{1,053}\pm 192~\mathrm{kcal}\cdot {\mathrm{BW}}_{\mathrm{kg}}^{0.75}$ per day (Orr, 1966; Hinchcliff et al., 1997).

Historical research has shown that breed differences affect energy requirements. The effect of breed differences can clearly be seen when comparing the energy requirements of Newfoundlands and Great Danes. Middle aged Newfoundlands (3–7 years old) have a MER of $106\pm 26~\mathrm{kcal}\cdot {\mathrm{BW}}_{\mathrm{kg}}^{0.75}$ (Rainbird & Kienzle, 1990) while Great Danes have a MER of 200–$250~\mathrm{kcal}\cdot {\mathrm{BW}}_{\mathrm{kg}}^{0.75}$ (Zentek & Meyer, 1992). In this study, breed did not appear to influence MER, but only a small population was studied. When the MER of Labrador retrievers was examined separate from the remaining breeds, their mean MER was $106\pm 36~\mathrm{kcal}\cdot {\mathrm{BW}}_{\mathrm{kg}}^{0.75}$ and was consistent with a previous study showing the MER of older laboratory Labrador Retrievers (>7 years) being $104\pm 32~\mathrm{kcal}\cdot {\mathrm{BW}}_{\mathrm{kg}}^{0.75}$ (Rainbird & Kienzle, 1990). A study evaluating a larger population would be important to determine if shepherds would have a different MER than Labrador retrievers.

The mean age of this population was 7.5 ± 2.4 years. While age was not associated with energy requirements in this study, most dogs were older than six years of age. An evaluation of a population including higher numbers of young dogs may be needed to determine the impact of age on this population’s maintenance energy requirements. Previous research has shown that MER in Labrador Retrievers, Great Danes and Beagles decreases with age (Kienzle & Rainbird, 1991).

While not analyzed, the effect of environmental temperature is unlikely to play a significant role in this population’s energy requirement. It is known that temperatures outside of the thermoneutral zone of 20–30 °C will increase energy requirements by 1–$5~\mathrm{kcal}\cdot {\mathrm{BW}}_{\mathrm{kg}}^{0.75}~\mathrm{per}~\deg \mathrm{C}$ per day when above or below this zone (National Research Council (NRC), 2006). During the study period, the dogs spent most of their time in the thermoneutral zone. The average low temperature dropped below the thermoneutral zone during 4 out of 6 months studied, but the dogs were active when outside during these temperatures and were housed indoors at night when daily temperatures were the lowest. The body heat generated from activity would have helped prevent additional energy expenditure to maintain body temperature when working in cooler weather (National Research Council (NRC), 2006). Due to the kennel’s geographic location, it is unlikely that indoor temperatures at night dropped below the thermoneutral zone despite low outdoor temperatures. However, the lack of records indicating the exact daily indoor temperature is a limiting factor in this study. If the study period had occurred in a climate where average daily temperatures were above the thermoneutral zone or remained below the thermoneutral zone for the entire day, an impact on energy requirements would require consideration.

Since there is a lack of data regarding the energy requirements of working dogs, it is difficult to apply this data to other forms of activity such as search and rescue or police dogs. In addition, other dogs with similar jobs may not perform the same amount of searches each month and may subsequently have different energy requirements. The evaluation and inclusion of other working dog facilities would help determine the appropriateness of applying these results to all working dogs.

While this is the first study to look at the energy requirements of working dogs, several limitations were present. This study was retrospective and the authors did not dictate the amount of food to be fed to each dog or the way the food was to be measured. This facility used measuring cups when determining feeding amounts. This feeding method has been proven to be imprecise, and weighing the food would have been more accurate (German et al., 2011). The authors also had no control over the breed and sex distribution of the dogs used at this facility. However, shepherd breeds and Labrador retrievers are commonly used in this line of work. In addition, the number of hours worked and the number of searches performed each day may not accurately assess the distance the dogs traveled during their jobs. GPS tracking devices or accelerometers (Wrigglesworth et al., 2011) could be considered in future studies to assist in accurately assessing daily activity levels. However, GPS and other tracking devices were not always allowed in the secure facilities where searches occurred. Finally, it would be beneficial to evaluate a full year of data to evaluate the effects of outdoor temperature extremes on energy requirements.

## Conclusions

While working dogs trained in odor, explosive and human detection are a vital part of securing facilities, no research had been published to document their energy needs. This study showed that the average energy requirement for this population of dogs was $136\pm 38~\mathrm{kcal}\cdot {\mathrm{BW}}_{\mathrm{kg}}^{0.75}$ or two times the calculated ideal weight RER. In addition, statistical analysis revealed a correlation between the number of searches a dog performed and the maintenance energy requirement. Approximately 8.5% of a dog’s maintenance energy requirement can be accounted for by the number of searches performed. Additional research is still needed to evaluate the energy requirements of dogs working in temperatures outside the thermoneutral zone and other types of working dogs such as search and rescue, police, and tracking dogs.

## Supplemental Information

10.7717/peerj.767/supp-1 Supplemental Information 1 Raw Data—Energy Requirements of Working Dogs Table shows the raw data from this study including body weights, energy intake, hours worked and number of searches.
